# Effects of novel anemia nurse manager program on hemodialysis: a retrospective study from Qatar

**DOI:** 10.5339/qmj.2021.46

**Published:** 2021-10-21

**Authors:** Abdullah Hamad, Hany Ezzat, Tarek Abdel Latif Ghonimi, Rania Ibrahim, Fatma Ramadan, Nadia Noor, Fadumo Yasin, Sahar Ismail, Fadwa Al-Ali

**Affiliations:** ^1^Hamad Medical Corporation, Doha, Qatar E-mail: ahamad9@hamad.qa

**Keywords:** anemia, end-stage renal disease, erythropoietin, hemodialysis, iron deficiency

## Abstract

Introduction: Anemia management in dialysis is challenging. Keeping hemoglobin levels within a tight range is difficult. A new program (anemia nurse manager [ANM]) was started for better anemia management. This study aimed to compare traditional anemia management with the new ANM model regarding the achievement of better hemoglobin targets (range, 10–12 g/dL), avoidance of extreme hemoglobin levels ( < 9 or >13 g/dL), and evaluation of the cost-effectiveness of the new model.

Methods: This retrospective observational study compared traditional anemia management with management involving our new ANM model. Patients on hemodialysis in all ambulatory dialysis clinics in Qatar were included. The study included three phases: phase 1 (observation): June 2015 to August 2015, 460 patients; phase 2 (pilot): September 2015 to May 2016, 211 patients; and phase 3 (expansion in two phases): June 2016 to February 2017 and October 2017 to June 2018, 610 patients. Hemoglobin, iron saturation, and ferritin were evaluated according to the protocol.

Results: In this study, 55% of the patients achieved the target hemoglobin in phase 1 compared with 75% in phase 2 (*p* = 0.0007). The hemoglobin level within the target range was sustained at 72% ± 5% of patients in phase 3. The achievement rate of the target hemoglobin level increased from 56% (May 2015) to 72% (July 2018) (*p* < 0.001). The proportion of patients with extreme hemoglobin declined from 10.7% in phase 1 to 6.4% in phase 2 and sustained at 8% afterward. Reducing the doses of erythropoietin stimulating agents, owing to the use of the ANM model, saved costs by approximately 11%.

Conclusions: The ANM model was able to achieve and maintain hemoglobin levels within the target range and decrease extreme hemoglobin levels. These outcomes improved patient care by avoiding high hemoglobin (increase thrombosis, cancer recurrence, stroke, and death) and low hemoglobin (weakness, poor quality of life, and need for transfusion) levels. The ANM model was cost effective even after including the salaries of nurses. This model can be considered in other aspects of patient care in dialysis.

## Introduction

Anemia is a common problem among patients with chronic kidney disease (CKD).^1^ Several mechanisms are involved including iron deficiency and decreased production of erythropoietin stimulating agents (ESAs).^2-3^ As CKD progresses to end-stage renal disease (ESRD), anemia tends to worsen, and management becomes more complicated. The World Health Organization defines anemia as hemoglobin (Hb) levels < 13 g/dL in men and post-menopausal women and < 12 in normal women^4^. Applying these targets in hemodialysis (HD) to improve the quality of life yielded mixed results.^5-7^ Large trials to normalize Hb levels in patients with anemia and ESRD on dialysis revealed increased stroke risk, mortality, recurrence of cancers, and lack of improvement in cardiovascular disease.^8-11^ These findings have led to a change in the Hb targets in patients with ESRD on dialysis. Recent guidelines have recommended maintaining the Hb level within 10–12 g/dL to prevent the need for blood transfusions and avoid complications.^12-14^

The Hamad General Hospital (HGH) is a comprehensive government healthcare facility that covers most healthcare needs for the State of Qatar. In Qatar, HD is provided through four ambulatory dialysis clinics under HGH (Fahd Bin Jassim Kidney Center [FBJKC], which included Wakrah (Area A) and Al-shamal and Al-shahania (Area B)). The mortality rate is relatively low in the younger population.^15^ Traditionally, anemia is managed in these centers by nephrologists. In 2015, HGH initiated a pilot program to evaluate the potential role of an anemia nurse manager (ANM) to improve anemia care in ambulatory dialysis centers. The idea originated from the fact that a dialysis nurse is readily available in the center and spends more time with the patients. The duties of the nurse include the following: review and update patients’ anemia-related medications and laboratory tests on file, review with the physician to write a prescription, follow up compliance and further tests, provide education to the patients and staff, and participate in multidisciplinary meetings and protocol updates. The ANM model began under the supervision of an experienced nephrologist who provided extensive training. This gradually expanded to include all ambulatory patients on HD in the State of Qatar. The model is different from the previous practice (which was dependent on the nephrologist, who has a busy schedule and thus limited availability) in that the new model employs a full-time ANM (available, trained, etc.) under limited supervision of a nephrologist.

**The primary aim** was to investigate the effect of the ANM model on achieving and maintaining Hb level within the target range (10–12 g/dL). The secondary aims were to study the effect of the model on avoiding extreme Hb levels ( < 9 g/dL or >13 g/dL) and investigate the cost-effectiveness of the new model.

## Methods

This retrospective longitudinal epidemiologic study was conducted to describe the effects of the ANM model on the Hb level of patients living in the State of Qatar. The population includes all ambulatory patients on HD in Qatar. The study received ethical approval from the Medical Research Center Institutional Review Board, Hamad Medical Corporation, Doha, Qatar (Approval No. MRC-01-18-266).

**Figure 1.**
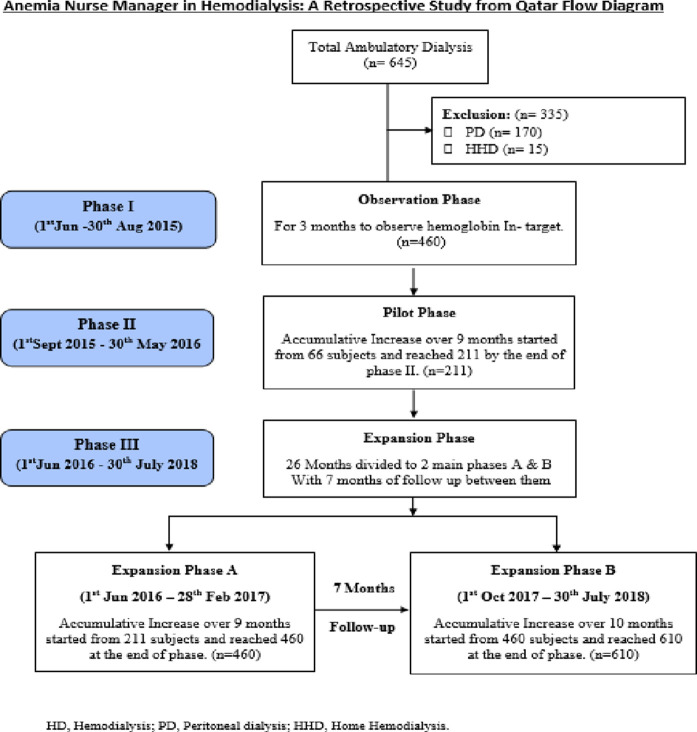
Effects of novel anemia nurse manager program in hemodialysis: A retrospective study from Qatar: Flow diagram

The study included patients aged >16 years with ESRD on HD for >1 month in an ambulatory HD clinic and excluded patients aged < 16 years or with ESRD on peritoneal dialysis. The study was performed in all ambulatory dialysis centers in Qatar (FBJKC Areas A and B). The study was completed in 3 years and 2 months (June 2015–July 2018), which was conducted in three phases. In phase 1 (observation phase), patients’ data were monitored for 3 months before the implementation of the program (June–August 2015) in all ambulatory HD units. In phase 2 (pilot phase; September 2015–May 2016), the ANM model was gradually implemented in Area A in FBJKC, whereas Area B received standard of care. In phase 3 (expansion phase; June 2016–July 2018), the ANM model was applied to all ambulatory dialysis centers in Qatar. It was divided into phase 3a with expansion to all FBJKC (June 2016–February 2017) and phase 3b (October 2017–July 2018) (Figure 1).

Collected data included patients’ characteristics, demographic information, comorbidities, outcomes, and clinical data, which were extracted from the electronic medical records. The focus was on the patients’ laboratory results relevant to the aims of this study (i.e., Hb, ferritin, and iron saturation levels), medications (ESAs including epoetin alfa, darbepoetin alfa, and methoxy polyethylene glycol-epoetin beta), and iron therapy (intravenous and oral), with attention to the doses, frequency of injections, and associated costs of treatment.

**Figure 2.**
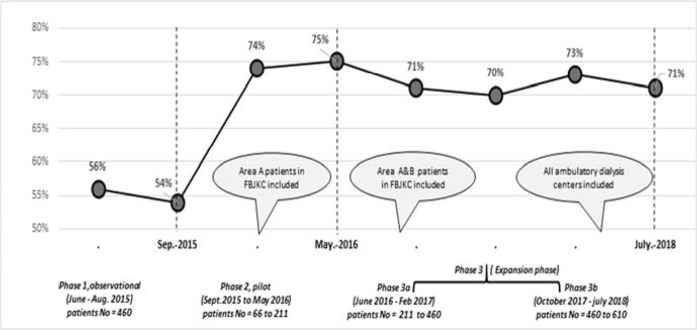
Percentage of patients on hemodialysis with target hemoglobin level (10–12 g/dL) during the study period according to phases.

The percentage of patients with Hb within the target range (10–12 g/dL) every month was calculated from the total number of patients with HD with Hb measured for that month (e.g., patients who were on prolonged hospitalization and whose Hb level was not measured in the ambulatory clinic were not included that month) (Figure 2).

A pharmacoeconomic analysis was performed because the health care in Qatar is generally almost completely subsidized by the government (including HD treatment and medications). The calculation was based on a simple formula: cost of medication (based on the purchase price paid by the hospital's central pharmacy) ×  number of units of each ESA used – salaries of nurses (calculated by the percentage of time allocated to work as an ANM).

### Statistical analysis

The Statistical Package for Social Sciences version 17.0 for Windows (SPSS Inc., Chicago, IL) was used in the data analysis. Continuous variables are presented as means ±  standard deviation or medians and ranges, and categorical variables are presented as absolute and relative frequencies. For comparison between groups, the paired t-test was used for parametric variables and the chi-square test was used for nonparametric variables. Probability values of *p* < 0.05 (two-tailed) were considered significant.

## Results

### Study population

The patient census during the study period increased from 460 in June 2015 to 610 in July 2018. Characteristics, demographics, and comorbidities for all accumulative patients through all phases are summarized in Table 1; no differences were found in comparing these variables among the three phases. The patients are relatively young, varied, and have multiple comorbidities. Members of the population have mixed ethnic backgrounds: 83.4% Middle Eastern (predominantly from Qatar and Egypt), 11.6% South Asian (mainly from India and Pakistan), and 4.9% East Asian (mainly from the Philippines). There is a high prevalence of hypertension (93%), diabetes mellitus (69%), and cardiovascular disease (41.5%). Table 1 summarizes the demographics, characteristics, and comorbidities of the study population.

### Primary aim

#### Phase 1 (observation phase)

The percentage of HD patients with Hb within the target levels ranged from 56% in June 2015 to 54% in August 2015 (mean, 55 ± 2).

#### Phase 2 (pilot phase)

The ANM model was initiated at FBJKC in September 2015 with 66 patients and gradually reached 211 patients by May 2016 (which included all patients in Area A at FBJKC). The percentage of patients with Hb within the target range of 10–12 g/dL steadily improved from 54% in September 2015 to 75% in May 2016 (*p* = 0.0007). The number of patients within the target range in the ANM model (Area A at FBJKC, n = 211), when compared with the standard of care (Area B at FBJKC, n = 147) in the 3-month period (May–July, 2016) (70 and 59%, respectively), was significantly different (*p* < 0.05).

#### Phase 3 (expansion phase)

The ANM model was gradually in all patients at FBJCK (460 patients in Areas A and B) between June 2016 and February 2017. In October 2017, the ANM was implemented to include all ambulatory dialysis clinics in Qatar (Alwakra, Alshamal, and Alshahania, with a total of 610 patients). Ninety-five percent of patients received ESA during the study period. The type of ESA used was changed over time based on administrative decisions (59% with epoetin alfa and 37% darbapoetin alfa in 2015 compared with 20% and 80% in July 2018, respectively).

The proportion of patients with Hb within target levels was sustained at 72% ± 5%. A comparison of the entire HD population revealed a remarkable increase in the percentage of patients within target levels (10–12 g/dL) from June 2015 (56%) to July 2018 (71%) (*p* < 0.001).

Moreover, a comparison of the 3-month averages within the Hb target levels before the introduction of the ANM model (phase 1, observation phase, June–August 2015) and the final 3-month period after the implementation of the model (from May 2018 to July 2018) revealed an increase in the percentage of patients within the target Hb range from 58% to 73%, respectively.

### Secondary aims

#### Extreme Hb level

The number of patients with extreme Hb levels decreased from 10.7% in September 2015 (phase 1) to 6.4% in May 2016 (phase 2) (adjusted for ESA-naïve patients).

In phase 3 (both 3A and 3B), low percentages of extreme Hb level were maintained with a mean of 8% of ambulatory HD patients (without adjusting for ESA-naïve patients).

The percentage of HD patients with ferritin levels of 200–800 mcg/L also improved between phases 1 and 2, increasing from 55% to 69% (*p* = 0.005), without a significant change in iron saturation. These findings persisted in phase 3.

#### Cost-effectiveness

Doses of ESAs reduced with the implementation of the ANM model. This reduction was replicated with every expansion phase. Cost analysis was performed using a simple formula. First, we calculated the number of patients on each type of ESA each month. Then, we estimated the cost savings for each ESA separately (darbepoetin savings were estimated at 10 US dollars) (USD) per week per patient (the average weekly dose per patient decreased from 46 to 41 mcg). For epoetin, the cost saving was 5 USD per week per patient (the average weekly dose decreased from 12000 IU to 10500 IU) compared with baseline. This translated into annual savings of approximately 158000 USD for darbepoetin and 50000 USD for epoetin (calculated for average census of 535 patients annually). By subtracting the nurses’ annual basic salaries (estimated at 56000 USD), an annual cost saving was over 150000 USD.

## Discussion

Renal anemia is a predictor of cardiovascular risk (He et al., 2017),^16^ and it usually develops because of erythropoietin deficiency, often compounded by iron deficiency. Several randomized trials (Drüeke et al., 2006; Pfeffer et al., 2009; Singh et al., 2006)^17-19^ have raised safety concerns about the normalization of Hb level using ESA; thus, international guidelines recommended the target Hb levels of 10–12 g/dL (KDIGO, 2012 (13); The Renal Association, 2017).^20^ Normalizing the Hb levels within these narrow targets using ESA is challenging, as several strategies evolved, including the use of protocols/algorithms. Maintaining the Hb level within the target range (10–12 g/dL) was challenging and reflected the difficulty in maintaining target levels within a narrow range over time, which can be complex and time consuming (Gardiner et al., 2019).^21^

The ANM proved to be a successful cost effective model with favorable outcomes. These outcomes were reproducible with every expansion phase. This approach is unique in the Middle East area. The program was initiated with two part-time nurses under the supervision of an experienced nephrologist who provided extensive training. We gradually extended the program to include all patients on ambulatory dialysis in the State of Qatar. The ANM reviewed the laboratory results, and the prescriptions for ESAs and iron were written simultaneously. The role of ANM in the program was similar to other experiences reported previously. In 2014, Gerrish^22^ reported that the prescriptive authority empowers the anemia nurse to review blood values and alter and prescribe medications accordingly, including changes outside the anemia protocol to use the lowest possible maintenance dose and thereby achieve the target Hb level. The prescriber is also responsible for transcribing the ESA dosage of patients who just started ESA therapy, where appropriate, to ensure continuity of care.

As advantages, the ANMs are available, can respond swiftly, and provide complete care, including reviewing the patients’ anemia-related medications, updating the patients’ file appropriately, monitoring patients’ compliance to medications, providing education, as well as involving family members and caregivers in the care plan, and coordinating patient care with other members of the multidisciplinary team (such as physicians, dietitians, social workers, and hematology experts) as necessary. This new model was cost effective, as the ESA doses required to maintain the Hb within target levels were reduced. This was achieved by avoiding overdosing and limiting wastage.

A comparison of the entire HD population revealed a remarkable increase in the percentage of patients within the target Hb level (10–12 g/dL) from June 2015 (56%) to July 2018 (71%) (*p* < 0.001). This finding is in agreement with the finding of another other study conducted by Yong and Kairaitis (2010),^23^ who reported a significant improvement in the proportion of Hb values within the target range after the implementation of a nurse-led protocol which had a higher Hb target range (11–13 g/dL) than that used in our study (10–12 g/dL). In 2020, George and McCann^24^ did not found significant differences in serum Hb levels and ESA dosages between pre- and post-nurse prescriber implementation protocol. However, they found a significant improvement in serum ferritin and transferrin saturation levels, and significantly less intravenous iron was required.

Our anemia management model was safe (by avoiding extreme Hb levels and maintaining Hb within the target range). The results were comparable with those of another study conducted by Drennan et al.^25^ who audited and researched nurse prescription authority and concluded that nurse prescriber prescriptions were appropriate and safe.

The ANM model was more successful in achieving the anemia targets compared with our old model likely because ANMs have more availability (close follow up of laboratory tests, medication adjustment was performed promptly, and strict compliance with dialysis anemia management protocol compared with physician-led management). Health care cost was reduced because Hb levels were maintained within the target range; having a stable Hb level led to few adjustments of ESA doses (especially when trying to overcorrect a low Hb level).

The implementation of our new anemia management model was challenging. However, logistic difficulties were overcame by providing continuous education and maintaining communication among doctors, pharmacists, nurses, and patients. This enables the smooth transition to the new management model. Moreover, having the nephrologists and staff accept the new model was a great challenge. Providing new ideas and learning the management processes and methods took a heavy discussion with other team members. However, continuous learning and support from the administration made it easier to implement and adapt the model. Complex cases were addressed with multidisciplinary collaboration, early management, and proper referral.

This study has some limitations. This retrospective cohort study used limited data and analysis (although most of the data were available in the electronic medical record; nationwide for the study duration). In addition, the comparison of anemia outcomes with baseline values has included some but not all the confounding factors that affect these values.

## Conclusion

This study revealed that the involvement ANM in the management of anemia in patients on dialysis led to the improvement in maintaining the target Hb level and reduced extreme Hb level. This would avoid short-term (blood transfusion, weakness, and access thrombosis) and long-term complications (stroke, cancer recurrence, poor quality of life, and death). The ANM model was cost effective even after factoring in the salaries of the nurses. The success of the ANM program is depended on the availability of the ANMs, adherence to the protocol, and empowerment. The study highlights the potential role of nurse prescribers in the continuity of care in dialysis.

### Acknowledgments

The authors profusely thank the Medical Research Center - Hamad Medical Corporation for their ethical and financial support. We would like to thank Editage (www.editage.com) for English language editing.

### Publication statement

This manuscript has not been published elsewhere in part or in entirety and is not under consideration by another journal.

### Prior presentation

Parts of this work were presented as a poster at the American Society of Nephrology, San Diego, CA, October 23, 2018, and won the Rising Star Award for project “Nursing Care Beyond Tradition,” Star of Excellent Award, Hamad Medical Corporation 2020.

### Funding statement

The study had been funded by the Medical Research Centre - Hamad Medical Corporation with the Ethical Approval No. MRC 0118266 on February 4, 2019.

### Competing interest statement

No potential conflicts of interest relevant to this article were reported.

### Data sharing

All the data collected during the study is presented in this manuscript and no data from the study has been or will be published separately. We attest that we will provide the data if requested according to HMC policy.

## Figures and Tables

**Table 1:** Demographic, characteristics, and comorbidities of the study population

Variable	No (%)

	610 (100)

Age (years)	

< 30	36 (5.9)

30– < 40	57 (9.3)

40– < 50	91 (14.9)

50–65	224 (36.7)

>65	202 (33.2)

Sex	

Male	361 (59.1)

Female	249 (40.8)

Ethnic background	

Middle East	509 (83.3)

South Asia	71 (11.6)

East Asia	30 (4.9)

Dialysis modality	

HD	610 (76.7)

PD	170 (21.4)

HHD	15 (2)

Comorbidities	

CAD	158 (25.9)

CVA	28 (4.6)

PVD	48 (7.8)

AF	20 (3.2)

Hypertension	567 (93)

Diabetes mellitus	408 (67)

